# Effects of Traditional Orchard Abandonment and Landscape Context on the Beneficial Arthropod Community in a Mediterranean Agroecosystem

**DOI:** 10.3390/insects14030277

**Published:** 2023-03-10

**Authors:** Víctor de Paz, Josep D. Asís, Andrea Holzschuh, Laura Baños-Picón

**Affiliations:** 1 Departmento de Biología Animal, Ecología, Parasitología, Edafología y Química Agrícola, Facultad de Farmacia, Campus Miguel de Unamuno s/n, Universidad de Salamanca, 37007 Salamanca, Spain; victordepaz177@usal.es (V.d.P.); lbanos@usal.es (L.B.-P.); 2 Department of Animal Ecology and Tropical Biology, Biocenter, University of Würzburg, Am Hubland, 97074 Würzburg, Germany; andrea.holzschuh@uni-wuerzburg.de

**Keywords:** abandonment, traditional almond orchard, spider, parasitoid, bee, landscape complexity

## Abstract

**Simple Summary:**

Agricultural abandonment is a very relevant process in Europe and its consequences on biodiversity are not yet clear. Moreover, abandonment is focused on marginal areas, characterized by traditional agricultural landscapes, with high cultural and biodiversity values, which are particularly vulnerable to this process. Although increasing attention has been paid to agricultural abandonment in recent years, studies focused on traditional orchards are scarce. In our study, we analyzed how abandonment and landscape complexity (measured as the percentage of semi-natural habitats) affected three groups of beneficial arthropods (spiders, bees and hymenopteran parasitoids) in almond orchards established on the border between Spain and Portugal. Our results showed that traditional and abandoned orchards harbor different arthropod communities, with the latter favoring these beneficial arthropods in simple landscapes (with a low percentage of semi-natural habitats). However, abandoned orchards lose relevance as the percentage of semi-natural habitat in the landscape increases, highlighting the important role that these habitats play in traditional agricultural landscapes. While abandoned orchards provide valuable habitats and alternative resources, they do not substitute semi-habitats nor traditional orchards. From a conservation management perspective, both traditional almond orchards and semi-natural habitats should be conserved to protect the rich biodiversity associated with these traditional cultural landscapes.

**Abstract:**

Agricultural abandonment is one of the main land-use changes in Europe, and its consequences on biodiversity are context- and taxa-dependent. While several studies have worked on this topic, few have focused on traditional orchards, especially in different landscapes and under a Mediterranean climate. In this context, we aimed to determine the effects of almond orchard abandonment on the communities of three groups of beneficial arthropods and the role of the landscape context in modulating these effects. Between February and September 2019, four samplings were carried out in twelve almond orchards (three abandoned and three traditional (active orchards under traditional agricultural management) located in simple landscapes as well as three abandoned and three traditional in complex landscapes). Abandoned and traditional almond orchards harbor different arthropod communities and diversity metrics that are strongly conditioned by seasonality. Abandoned orchards can favor pollinators and natural enemies, providing alternative resources in simple landscapes. However, the role that abandoned orchards play in simple landscapes disappears as the percentage of semi-natural habitats in the landscape increases. Our results show that landscape simplification, through the loss of semi-natural habitats, has negative consequences on arthropod biodiversity, even in traditional farming landscapes with small fields and high crop diversity.

## 1. Introduction

Habitat loss by conversion to intensive agriculture constitutes one of the main drivers of biodiversity loss worldwide, and it has been identified as the primary cause of the decline in insect populations [1,2,3,4]. For these reasons, the consequences of agricultural intensification (e.g., landscape simplification; farmland consolidation; the increased use of synthetic pesticides, herbicides, and fertilizers; habitat fragmentation; a reduction in crop diversity) and the possible measures to counteract them have been thoroughly investigated [5,6,7,8,9].

In Europe, while intensification is still the main trend on fertile soils, agricultural abandonment is prevalent in marginal areas with poor soils; in fact, the total area dedicated to agriculture has been steadily decreasing over the last few decades [10,11]. The consequences of agricultural abandonment on biodiversity are still subject to much controversy. While some studies show an opportunity for the restoration of natural ecosystems [12], others point out the loss of the rich biodiversity associated with traditional farming systems [13,14,15]. Many studies have attempted to shed light on this topic, and the rough conclusion seems to be that in Europe, agricultural abandonment reduces overall biodiversity, especially in traditional landscapes and after long periods [11,16,17]. Conversely, some studies have found increased diversity in late successional stages or an overall beneficial effect of maintaining both managed and abandoned plots in the landscape [18,19,20,21,22,23].

The majority of the studies on the effects of farmland abandonment have focused on grasslands or annual crops, especially in central Europe, whereas permanent crops and, in particular, traditional orchards, have received far less attention [24,25]. As ecological theory predicts, the consequences of agricultural abandonment (i.e., a further reduction in the disturbance level) may not be the same in annual systems and permanent crops given the low disturbance levels and increased stability of the latter [26]. This is based on the *intermediate disturbance hypothesis*, which predicts higher diversity levels at intermediate levels of disturbance, with both poorly and highly disturbed systems harboring less biodiversity [27,28]. Furthermore, the results observed in central Europe may differ in Mediterranean areas given the extreme changes that ecosystems suffer under this climate, with a strong variability in resource availability between seasons [29]. This severe seasonality can affect plant and arthropod communities and could interact with crop management changes [30,31].

Studies assessing the consequences of agricultural abandonment in different landscape scenarios are even scarcer, notwithstanding that these consequences may be modulated by the landscape context, differing between simple and complex landscapes (*intermediate landscape complexity hypothesis* [32]). Therefore, interactive effects between landscape complexity (measured using the percentage of semi-natural habitats in the landscape as a proxy of complexity) and crop management practices (or their absence) at the local scale should be expected. In addition, within a landscape, habitat complexity affects biodiversity (see [33], and references therein). Plant diversity and vegetation architecture increase habitat complexity and have been proven to positively affect natural enemies [33,34,35,36] and pollinators [37,38,39] through a rise in niche and resource availability as well as diverse plant volatiles [40,41].

Consequently, the importance of semi-natural habitats depends on both occurrence in the landscape and habitat features. They provide different microhabitats and resources (i.e., floral resources, shelter, alternative hosts or prey, overwintering sites) that favor natural enemies and pollinators, which can, in turn, spill over into crops [32,42,43,44,45,46]. In fact, their relevance is such that Duelli and Obrist [47] found that more than 63% of all animal species inhabiting an agriculturally managed area depended on the presence of semi-natural habitats, although this dependence seems to be stronger in annual than in permanent crops [48,49,50].

Among permanent crops, traditional orchards constitute high nature value systems that, in Europe, have been managed in the same way for centuries, creating cultural landscapes with high levels of associated biodiversity that depend on their traditional farming practices to persist [51,52,53,54]. Almond orchards are among the best examples of high nature value systems, with the almond being an ancient crop that has been managed in the Mediterranean Basin since the Phoenicians introduced them by 300–500 BCE [55]. Within the Mediterranean region, Spain is the second largest producer in the world with 416,950 tons of almonds [10]. Traditional almond orchard yields depend on the delivery of ecosystem services by beneficial arthropods since almond trees need insect pollination to achieve fruit sets and are highly sensitive to various pests, benefiting from natural pest control [56,57,58].

Accordingly, this study focuses on three relevant groups of beneficial arthropods that interact with almond crops. Wild bees constitute one of the most important groups of pollinators, and they play a pivotal role in almond pollination [58,59,60]. Spiders are one of the most abundant and diverse groups of generalist predators inhabiting agroecosystems, with an important role in pest suppression, and have been found to dominate the natural enemy community in the almond tree canopy [61,62,63]. Hymenopteran parasitoids (hereafter parasitoids) are among the most relevant groups of natural enemies, being key for the control of almond orchard pests [64,65,66]. These groups also require a variety of resources (e.g., diverse vegetation structure and prey availability for spiders, flower resources and hosts for parasitoids, flower resources and nesting sites for bees) and can, therefore, act as indicators of the availability of these resources in different habitats [35,37,39,44,67,68].

Despite the importance of almond crop production, to our knowledge, no studies have evaluated the effects of almond orchard abandonment on their associated arthropod biodiversity, especially in traditional areas under different landscape contexts and in a Mediterranean climate with strong seasonality. To contribute to filling this knowledge gap, we set out to determine the following: (1) the possible differences in community composition, abundance, and richness of spiders, parasitoids, and bees between abandoned and active orchards under traditional management (hereafter traditional orchards); (2) the role of the landscape context in which our traditional and abandoned orchards are located in modulating these effects; and (3) the response of spiders, parasitoids, and bees to land-use composition at different spatial scales (150 m and 500 m). Based on this framework of knowledge and previous research, we hypothesized that the study groups’ community structure and composition will differ between managed and abandoned orchards, with more mobile organisms (i.e., bees) being more influenced by the landscape context than by the system. We also expect a generally positive effect with an increase in the percentage of semi-natural habitats in the landscape regardless of the system. Furthermore, we anticipate marked seasonality effects influencing the responses to orchard systems and semi-natural habitats through changes in resource availability.

## 2. Materials and Methods

### 2.1. Study Area

The study was carried out in the municipality of La Fregeneda (Salamanca, Western Spain) (40°59′ N, 6°52′ W). The location of this region, on the border with Portugal, together with the presence of small villages and limited infrastructures, has led to progressive isolation and depopulation as well as to a growing abandonment of agricultural activities. This socioeconomic situation has been a factor shaping the landscape, resulting in a mosaic of traditionally cultivated plots (with little mechanization and minimal economic investment) and abandoned plots in various stages of plant succession, interspersed with fragments of natural vegetation. These remnants of natural vegetation typical of Mediterranean sclerophyllous scrub are formed mainly by *Cytisus* shrublands (*Cytisus multiflorus* (L’Hér.) Sweet, *C. scoparius* (L.) Link), rockroses (*Cistus ladanifer* L.), French lavender (*Lavandula pedunculata* (Mill.) Cav.), and thymes (*Thymus mastichina* (L.) L., *Thymus zygis* subsp. *zygis* Loefl. ex L.), among others. There are also areas of Mediterranean forest, mainly composed of holm oaks (*Quercus ilex* subsp. *ballota* (Desf.) Samp.), although they coexist with European nettle trees (*Celtis australis* L.) and junipers (*Juniperus oxycedrus* L.), which have great ecological value and persist mainly because of the difficulty in cultivating on the steep slopes of the area, which has considerably limited agricultural practices.

**Figure 1:**
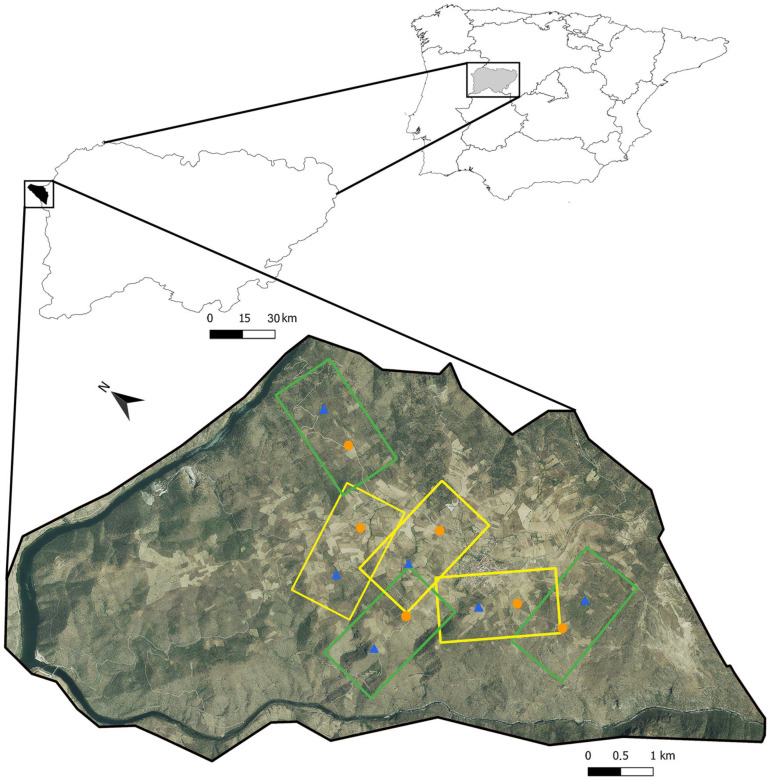
Location of the study area in the Salamanca province (Western Spain) and the placement of the 6 landscape windows (yellow: low level of semi-natural habitats, green: high level of semi-natural habitats) and 12 almond orchards (blue triangles: abandoned almond orchards, orange circles: traditional almond orchards).

The study area comprises 4900 ha, its altitude ranging between 130 and 560 m a.s.l. It is located within the Arribes del Duero Natural Park and belongs to landscape unit 84, “Gorges and valleys on the Portuguese border”, with a small part located in the transition zone between this and landscape unit 49, “Peneplains of Zamora and Salamanca and foothill of the Montes de León” [69] (Figure 1). The climate is mild, warm, and temperate, with an average annual rainfall of 626 mm and an average annual temperature of 13.7 °C. Numerous small watercourses run through the area, which is also crossed by the CL-517 along 12.8 km. The predominant soil types are eutric cambisol and lithic leptosol [70], following the World Reference Base for Soil Resources [71]. Attending to habitat composition and configuration, two types of landscapes can be differentiated in the study area: a complex one, with a high proportion of semi-natural habitats (mainly scrublands but also forest remnants and ungrazed grasslands) distributed in medium-to-large patches (0.5 to 20 ha), with inserted clusters of agricultural fields; and a simpler one, closer to the village, dominated by crops (mainly almond orchards, although olive groves, vineyards, cereal fields, and grazed grasslands are also present) interspersed with small-to-medium patches (0.01–1 ha) of semi-natural habitats (grasslands and scrublands that are no longer grazed, woodland hedges, road verges, and abandoned orchards that have reached late successional stages). These crops are traditionally managed without the application of synthetic pesticides or fertilizers. The orchards still active are dominated by old trees of indigenous varieties that are not significantly profitable and are owned by small producers who sell the harvested product to cooperatives in other regions, dedicating part of the production to self-consumption. However, the social and economic revitalization of this municipality depends largely on this crop since the flowering season represents its main tourist attraction.

### 2.2. Sampling Design

We selected six landscape windows varying in their percentage of semi-natural habitats, three of them with a low percentage of semi-natural habitats (mean 19.91 ± 11.68 %, low level of semi-natural habitats) and the other three with a high percentage of these habitats (60.19 ± 2.69 %, high level of semi-natural habitats). Each window was 1.85 km by 1 km in size—this was the minimal distance that allowed for us to select an abandoned almond orchard and an active one, under traditional agricultural management, always keeping a minimum distance of 500 m between orchards and between them and the window’s edges. Landscape windows allowed for us to standardize orchard selection, ensuring that each pair of traditional and abandoned orchards was located in the same landscape. The selected traditional orchards had a mean size of 6530.3 ± 3036.8 m^2^ and were rainfed, plowed once or twice a year to control the naturally growing vegetation, and pruned once a year; synthetic pesticides, herbicides, and fertilizers were not applied. The vegetation growing in these orchards was dominated by grasses such as *Festuca* sp., *Hordeum* sp., and *Avena* sp. and by Asteraceae; *Carduus* sp. and *Verbascum pulverulentum* Vill., among others, were also present. Selected abandoned orchards had a mean size of 6456.9 ± 1395.4 m^2^ and a similar vegetation structure, to guarantee that all of them were in a similar successional stage, that was composed predominantly of shrubs (*Cytisus multiflorus*, *C. scoparius*, and *Lavandula pedunculata*), *Festuca* sp., and Asteraceae, among others. We also measured the land-use composition in circular sectors with 150 m and 500 m radii surrounding each orchard, with five land-use categories (artificial roads and buildings, semi-natural habitats, permanent crops, grasslands, and annual crops), using the data from the Spanish Geographic Information System for Agricultural Plots (SIGPAC).

Sampling was performed from February to September 2019 every seven-to-eight weeks, starting with the flowering period (late February) and finishing just after the almond harvest (mid-September). Each sampling period took place over six consecutive days, randomly assigning the order in which the orchards were sampled. Weather conditions were kept as uniform as possible between the sampling periods, avoiding rainy and windy days.

**Figure 2:**
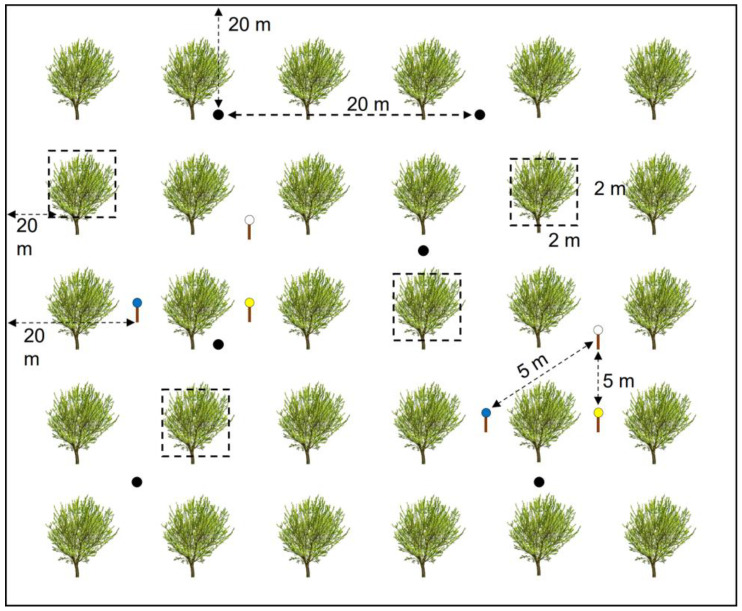
Sampling design. Black circles: pitfall traps; empty squares: 2 m × 2 m vacuuming quadrants; yellow, white, and blue circles: pan traps. Dashed arrows represent the minimum distance between traps or to the grove’s edge.

To capture spiders and hymenopteran parasitoids, six uncovered pitfall traps (9 cm diameter, 12.3 cm depth) were placed in each orchard, three under the almond tree canopy and three between rows (72 in total) [72]. The traps were filled to a third with a mixture of 70% alcohol and antifreeze (10% ethylene glycol) in a 3:2 ratio (600 mL of alcohol and 400 mL of antifreeze per liter). The traps were also placed 20 m apart from each other and the groves’ edges to reduce trap-to-trap interference and edge effects and they remained in the field for 72 h. To collect parasitoids and vegetation spiders, we randomly selected four trees in each almond orchard and vacuumed each tree and the surrounding vegetation in a 2 m × 2 m quadrant for three minutes using a gardener’s leaf blower (Garland GAS 550 G) modified as a suction machine [73]. For the capture of bees and parasitoids, we set up in each orchard two clusters of three pan traps each—one yellow, one blue, and one white. We used 500 mL plastic soup bowls painted with UV-bright yellow, white, and blue paint, and we placed them on iron poles at a 1 m height. Within each cluster, traps were placed 5 m apart. The traps were filled with water and a drop of detergent and were left in the field for 48 h [74] (Figure 2).

All the collected specimens were sorted in the laboratory and identified at either the family level (spiders and parasitoids) or the genus level (bees). A higher taxa approach (e.g., family or genus taxonomic resolution) was found to be a reliable approach for revealing species richness and compositional patterns [75].

### 2.3. Statistical Analyses

Prior to performing the analyses, we assessed the completeness of the sampling methods for each group (spiders, parasitoids, and bees) using the non-parametric Chao1 estimator [76]. All methods exhibited high levels of completeness (pitfall traps: 96% of the 24 estimated spider families, 93% of the 15 estimated parasitoid families; vacuuming: 83% of the 29 estimated spider families, 98% of the 24 estimated parasitoid families, 100% of the three estimated bee genera; pan traps: 96% of the 12 estimated spider families, 93% of the 21 estimated parasitoid families, 86% of the 22 estimated bee genera). We also removed the genus *Apis* (57 individuals) from the analyses to focus only on wild bees.

The effects of the *system* type (traditional vs. abandoned) and the *level of semi-natural habitats* in the landscape window (high vs. low) on the spider, parasitoid, and bee communities were analyzed with PERMANOVA (*system*, *level of semi-natural habitats*, and *sampling month* as fixed factors and *orchard* as a random factor, with 9999 permutations and “permutation of residuals under a reduced model” as the permutation method) and MDS (multidimensional scaling). Similarity matrices were calculated using the Bray–Curtis coefficients with the abundances square-root–transformed to reduce the weight of the most dominant families.

To test for spatial autocorrelation among the study orchards, we performed a Mantel correlogram based on a similarity matrix (Bray–Curtis) and the geographical data of the study orchards [77]. The results revealed a significant spatial autocorrelation for the bees and some of the spider and parasitoid families. Therefore, we added spatial correlation structures to these models and compared their AICs to select the best model [78]. We also checked for temporal correlation using the autocorrelation function (ACF), and when we detected significant temporal patterns, we added a correlation structure for short time-series and a variance structure that allows for different variances for each level of the variable *sampling month* to our models and compared the AICs [78] (Tables S1 and S2).

Family and genus richness and abundance data were analyzed in a two-step process. First, we used linear mixed models and generalized linear mixed models to test the effect of the *system* (traditional vs. abandoned), the *level of semi-natural habitats* in the landscape window (high vs. low), the *sampling month*, and their interactions (as fixed factors) and the *orchard* (as a random factor) on the family or genus richness and abundance for the spiders, parasitoids, and bees and for the abundance of the most dominant families or genera (>100 individuals, 20 out of 73).

Second, we checked the correlation between each land-use category measured at 150 m and 500 m, and since they were all negatively correlated with the percentage of semi-natural habitats at 500 m, we decided to use only this category in our analyses. We also checked the correlation between the percentage of semi-natural habitats and the system to ensure that neither abandoned nor traditional orchards had higher or lower levels of this variable and they were not correlated at either scale. To analyze the effect of semi-natural habitats at 150 m and 500 m on the pooled data from the four sampling periods, we used the linear least squares and generalized linear models, including the variables *system* and *percentage of semi-natural habitats*, at both scales (in separate models) and their interaction as fixed factors; then, we used the family or genus richness and the abundance of spiders, parasitoids, and bees and the abundance of the dominant families or genera as dependent variables. In both cases, we used the AICs to determine at which scale each group showed a stronger response [79].

To select the best models in all analyses, we used a standard backward selection procedure, and we checked the residuals of each model to ensure normality, independence, and homoscedasticity either graphically or with the package Dharma [80].

For the analyses, the statistical packages PRIMER v6 (PERMANOVA, MDS) (PRIMER-E Ltd., Plymouth, UK) [81] and R 4.0.5 (Mantel correlogram, ACF function, correlation matrices, linear least squares models, linear mixed models, generalized linear models, generalized linear mixed models) [82] were used.

## 3. Results

We collected a total of 8352 arthropods belonging to the focal groups: 4480 spiders (31 families), 3161 parasitoids (23 families), and 711 bees (20 genera).

**Figure 3:**
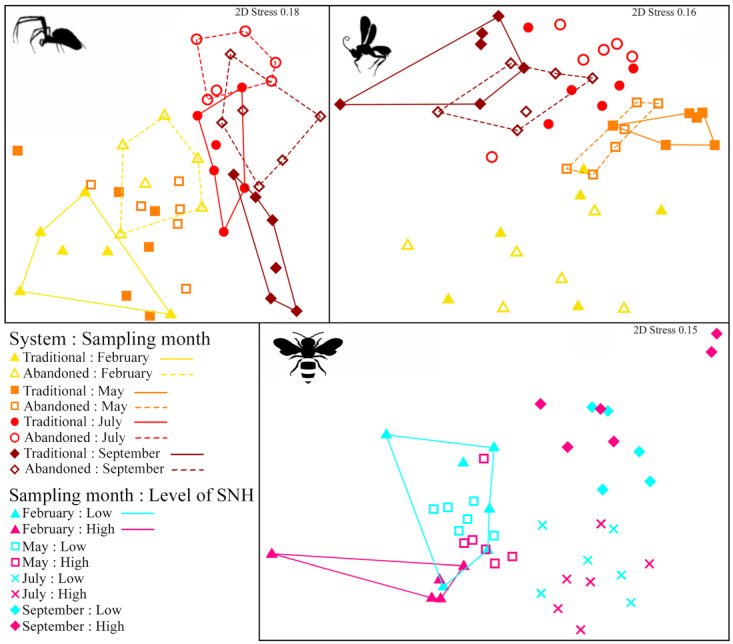
MDS for the spider, parasitoid, and bee communities sampled (Bray–Curtis index, square-root–transformed abundances). Polygons include the samples belonging to the same category of the variable system or level of semi-natural habitats for each sampling period. Only polygons for which significant pairwise comparisons were detected in the PERMANOVA are represented. SNH = semi-natural habitats.

The PERMANOVA revealed a significant effect of the *system* on the spider community (pseudo-F = 2.114, *p* = 0.003), with differences in every month except May, and on the parasitoid community (pseudo-F = 2.351, *p* = 0.0176), differing only in May and September. For the bee community, we found only a significant effect for the *level of semi-natural habitats* in February, right at the almond blooming period; this effect faded away as the season progressed (Table 1). These results are also noticeable in the MDS (Figure 3).

The linear models not only provided different results for each group but also showed some similar general trends, especially in the response of the different groups to the percentage of semi-natural habitats at both scales.

**Figure 4:**
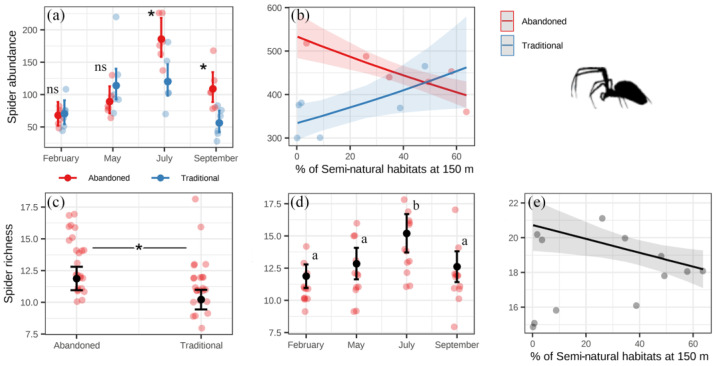
Results of the linear models for spider abundance (**a**,**b**) and richness (**c**–**e**). Points with error bars represent estimated means and 95% confidence intervals from the models’ predictions, and dull dots represent the raw data. Significant differences (*p* < 0.05) are shown with asterisks. Differences in pairwise comparisons are shown with different letters. ns: not significant.

Starting with the spiders, we found that their abundance was higher in abandoned than in traditional orchards toward the second part of the season (July and September; Figure 4a). Spider richness was higher in the abandoned orchards during the whole season (Figure 4c). Both spider abundance and richness peaked in July (Figure 4a,d, Table S1). Spiders were not affected by the level of semi-natural habitats in the landscape window (1.85 km × 1 km). Spider abundance decreased in abandoned orchards and increased in traditional orchards as the percentage of semi-natural habitats at 150 m increased (Figure 4b), and spider richness decreased as the percentage of semi-natural habitats at 150 m increased (Figure 4e, Table S2). Regarding spider families, gnaphosids, linyphids, and lycosids were more abundant in traditional orchards (in contrast to the results for all spiders), while araneids, oxyiopids, philodromids, salticids, and theridids were more abundant in abandoned orchards, and thomisids and zodarids were not affected by the system (Table S3). Some of them were affected by the level of semi-natural habitats in the landscape window (Table S3). Most spider families responded more strongly to the percentage of semi-natural habitats at 150 m than to other scales, in most cases showing the same interaction that we found for the whole group, with abundance decreasing in abandoned orchards and increasing in traditional ones as the percentage of semi-natural habitats increased (Table S4).

**Figure 5:**
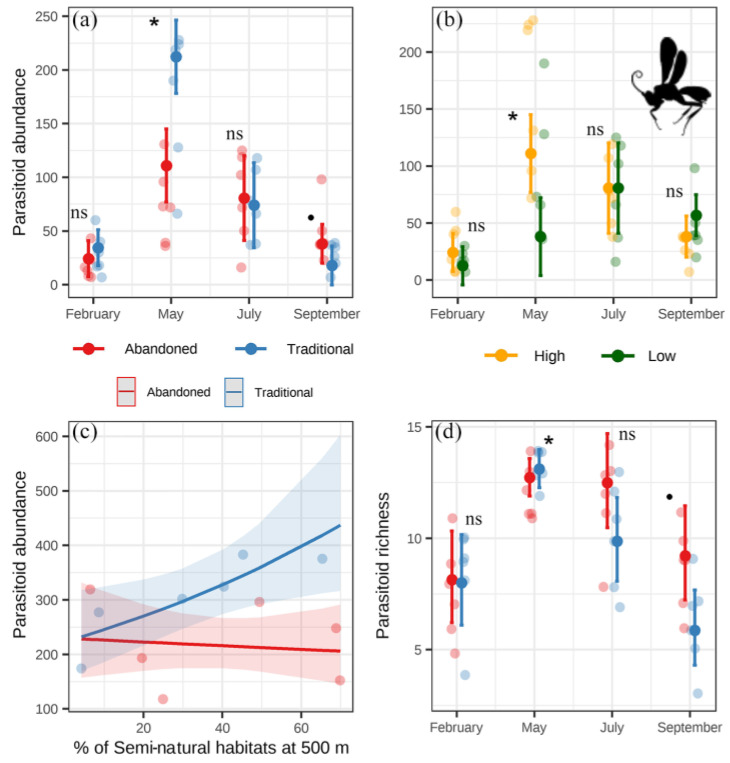
Results of the linear models for parasitoid abundance (**a**–**c**) and richness (**d**). Points with error bars represent estimated means and 95% confidence intervals from the models’ predictions, and dull dots represent the raw data. Significant (*p* < 0.05) differences are shown with asterisks and marginally significant (0.05 < *p* < 0.1) differences with dots. ns: not significant.

For the parasitoids, abundance and richness were higher in traditional than in abandoned orchards in May (when parasitoids had their peak) but were marginally higher in abandoned than in traditional orchards in September (Figure 5a,d). Only in May was their abundance significantly higher in orchards located in landscapes with a high rather than low level of semi-natural habitats (Figure 5b). In contrast, parasitoid abundance in traditional orchards increased as the percentage of semi-natural habitats increased at 500 m and decreased in abandoned orchards (Figure 5c, Table S2). Parasitoid richness was not affected by the percentage of semi-natural habitats at 150 m or 500 m. The dominant parasitoid families (Braconidae, Encyrtidae, Eulophidae, Mymaridae, Platygastridae, Pteromalidae, Scelionidae) were generally more abundant in traditional orchards and landscapes with a higher level of semi-natural habitats in May (Table S3), and they showed a stronger response to the percentage of semi-natural habitats at 150 m (except for pteromalids and braconids), usually maintaining the same interaction that we found for the whole group (Table S4).

**Figure 6:**
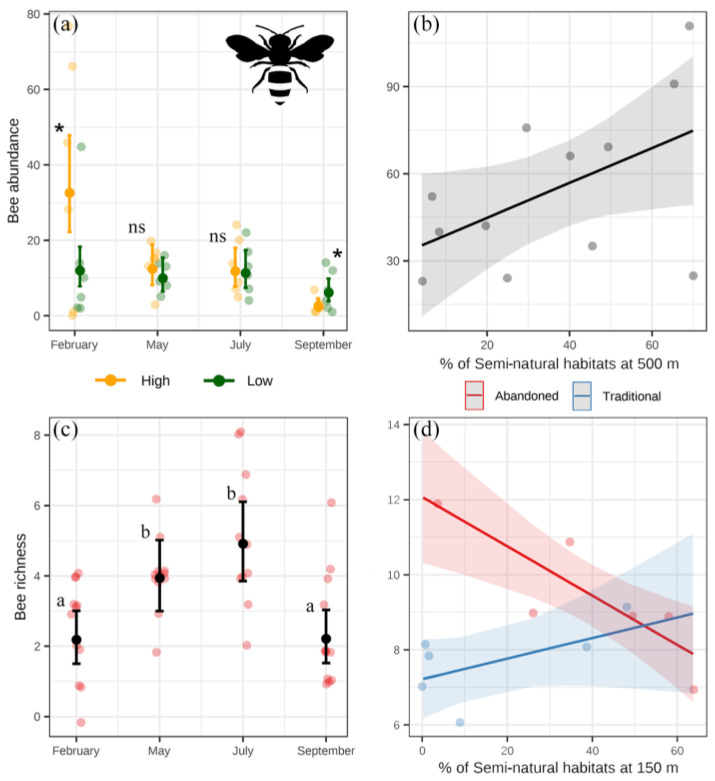
Results of the linear models for bee abundance (**a**,**b**) and richness (**c**,**d**). Points with error bars represent estimated means and 95% confidence intervals from the models’ predictions, and dull dots represent the raw data. Significant differences (*p* < 0.05) are shown with asterisks. Significant differences (*p* < 0.05) in pairwise comparisons are shown with different letters. ns: not significant.

For the bees, the highest abundance was found in February and the highest richness at mid-season (Figure 6a,c). Bee abundance in February was higher in orchards located in landscapes with a high level of semi-natural habitats (Figure 6a). Late in the season, bee abundance was higher in orchards located in landscapes with a low percentage of semi-natural habitats, but the number of individuals in this period was generally significantly low (Figure 6a, Table S1). Bee abundance increased with the percentage of semi-natural habitats at 500 m (Figure 6b), and richness decreased in abandoned orchards and increased in traditional ones as the percentage of semi-natural habitats increased at 150 m (Figure 6d, Table S2).

## 4. Discussion

Our results showed a strong effect of the system (abandoned vs. traditional) on the community composition, abundance, and richness of spiders and parasitoids, while no effect was found for bees. Bee and parasitoid abundances also responded to the level of semi-natural habitats in the landscape window (high vs. low), but spiders were not affected by this variable. Several metrics of the studied groups behaved differently in abandoned and traditional orchards depending on the percentage of semi-natural habitats in the surrounding 150 m or 500 m sectors. The strong seasonality of the Mediterranean climate had a decisive effect on all our results.

### 4.1. System Effects

In agreement with our hypothesis, abandoned and traditional almond orchards showed contrasting spider and parasitoid communities. These two types of orchards differ in vegetation architecture and microhabitats and resource availability (i.e., flowers, prey, hosts, refuge); therefore, they could be harboring species with distinct requirements. The spider community was richer and more abundant in abandoned orchards, which tend to have higher habitat structural complexity and resource availability, allowing them to sustain more species and individuals [25,33]. This is consistent with numerous studies that have shown that spiders are constrained by different habitat features at a local scale (e.g., [83,84]), being favored in habitats with increased prey availability [67,85] and more complex vegetation architecture [25,68,86,87]. In fact, only three families were more abundant in traditional orchards: lycosids, gnaphosids, and linyphids (ground runners and sheet web weavers [88]), which are favored by the more open habitat of traditional orchards [25,68,89,90].

Conversely, contrary to our expectations, at the beginning-middle of the season (May), parasitoid abundance and richness were higher in traditional orchards. In this period, traditional orchards have a diverse annual plant cover, more so than that of the shrub-dominated abandoned orchards; that plant cover does not dry out until early summer (late June), offering a great availability of floral resources and hosts, including almond pests, which could favor the presence and colonization of parasitoids from surrounding habitats [35,42,44,91].

Both parasitoid richness and abundance, as well as spider abundance, were higher in abandoned orchards exclusively in mid–late summer (July–September). Mediterranean ecosystems experience dramatic changes throughout the year, with strong disparity in resources availability between seasons and an expected reduction in plant and host accessibility during the summer drought [29,30]. The consequences of this severe seasonality are exacerbated in traditional almond orchards, where the annual vegetation dries up earlier than in the shrub-dominated abandoned orchards, reducing vegetation complexity and thus hampering the diversity of resources that benefit spiders (i.e., anchoring points, prey abundance [67,92]) and parasitoids (i.e., floral resources, alternative hosts, refuge [44,91]).

Bee richness was almost only affected by seasonality, reflecting a high species turnover caused by the marked changes that ecosystems suffer under a Mediterranean climate ([93], and references therein). The lack of differences in bee abundance and richness between abandoned and traditional almond orchards could be explained by the successional stage of our abandoned orchards since the shrubland stage, dominated by one or two *Cystus* or *Cytisus* species, was found to provide less food resources and harbored the lowest bee abundance and richness of any successional stage of those compared in a recent study in a similar ecosystem [18]. The absence of differences could also be derived from the main management practice performed in the traditional orchards (tillage), which has been found to negatively affect the ground nesting bees (such as *Andrena*, *Eucera*, *Halictus* or *Lasioglossum*) that dominate our community [94,95]. In consequence, it is likely that neither abandoned nor traditional almond orchards would provide sufficient feeding and nesting resources outside of the almond blooming period, explaining our generally low bee abundance.

### 4.2. Landscape Effects

Parasitoid abundance was higher in landscape windows with a high level of semi-natural habitats. Complex landscapes, with a high percentage of semi-natural habitats, have greater habitat heterogeneity than simplified agriculture-dominated landscapes, thus providing a wider variety of microhabitats, overwintering and feeding resources, and alternative hosts [32,36,96,97]. Diverse and abundant resources may result in increased parasitoid abundance in landscapes with a high percentage of semi-natural habitats [91,96,98,99,100,101]. Moreover, parasitoids, given the high trophic level, are particularly sensitive to landscape simplification since they are directly and indirectly affected by habitat loss and agricultural perturbations [102,103,104].

As we hypothesized, bees responded more strongly to the level of semi-natural habitats in the landscape window than to the system. In February, different and more abundant communities appeared in orchards located in landscapes with a high percentage of semi-natural habitats, and they also showed a general increase in abundance as the percentage of semi-natural habitats at 500 m increased. In these complex landscapes, semi-natural habitats are represented by medium-to-large patches (0.5–20 ha) of ungrazed grasslands, forest remnants, and predominantly shrublands, interspersed with clusters of agricultural fields. Semi-natural habitats provide nesting and alternative foraging resources that could limit bees, especially during the almond blooming period in February, when bee abundance peaks, since they need foraging resources both before and after bloom in other habitats because the blooming period might be too short even for wild bees with short life spans [37,38,39,94,105,106].

Unexpectedly, several metrics (spider abundance, parasitoid abundance, bee richness, and the abundance of the majority of the dominant families and genera) showed a similar response to the percentage of semi-natural habitats measured at 150 m or 500 m. Either abundance or richness decreased in abandoned orchards and increased in traditional orchards as the percentage of semi-natural habitats increased (generally at 150 m). To our knowledge, only one other study in Europe assessed the interactive effects of landscape complexity and traditional orchard abandonment on arthropod abundance or diversity, and contrary to our results, an interaction between landscape structure and management practice was not found [107]. Given this lack of similar studies, we can only hypothesize, based on our results and ecological theory [32,47], that in areas with a low percentage of semi-natural habitats, dominated by small agricultural fields with different land uses, abandoned orchards constitute favorable patches, providing alternative resources for foraging, nesting, and refuge. Therefore, they could act as islands, providing reservoir habitats in an unfavorable matrix. Nevertheless, as the percentage of semi-natural habitats escalates, habitat heterogeneity and resource availability increase, translating into higher complementarity and heightened arthropod movement between patches [9,32,96]. Within these conditions, abandoned orchards lose relevance, and the biodiversity that they harbor decreases.

On the other hand, in the traditional orchards located in areas with a low percentage of semi-natural habitats, the studied groups may be limited by management practices and the scarcity of alternative resources. This trend is observed despite the high crop heterogeneity and small field size, following the general pattern of European studies where other variables such as landscape complexity are of greater relevance ([108] and references therein). However, as the percentage of semi-natural habitats increases, these limiting capacities become available in the surrounding area, thus allowing the communities of the study groups to thrive and spill over from these unmanaged habitats and successfully exploit the resources (especially prey or hosts and floral resources during the blooming period and spring) that traditional orchards provide [32,42,109,110,111].

## 5. Conclusions

Agricultural abandonment is a relevant land-use change process driven by rural depopulation which, although relevant worldwide, is particularly prevalent in Spain and many other European countries. Agri-environmental payments (EPAC) have been focused on preventing the abandonment of traditional farming systems, which is considered one of the main drivers of biodiversity loss in Europe [112]. Nevertheless, research efforts to elucidate farmland abandonment consequences on biodiversity have been unequally distributed between annual and permanent crops, the latter being scarcely investigated [24], with only a few studies generally focused on olive groves, apple orchards, or vineyards, e.g., [20,23,25]. To our knowledge, our work constitutes the first attempt to analyze the effects of almond orchard abandonment on arthropod biodiversity, and it suggests that both abandoned and traditional almond orchards play an important role in conserving rich and abundant arthropod communities—but only in simple landscapes.

Arthropod community composition at the field scale is driven by orchard management, with abandoned and traditional orchards harboring different communities and diversity metrics that are strongly conditioned by seasonality (defined as the climatic differences between sampling periods (e.g., temperature, rainfall and humidity) that condition the plant community and influence arthropod assemblages).

The landscape context significantly interacts with almond orchard management. In our study area, abandoned orchards located in simpler landscapes seem to assume the role of semi-natural habitats in providing alternative resources that can favor pollinators and natural enemies. Nevertheless, these abandoned orchards do not substitute semi-natural habitats since their arthropod community diversity decreases as the percentage of semi-natural habitats in the surrounding area increases. Conversely, in traditional orchards, diversity increases with landscape complexity, reflecting the relevant role of semi-natural habitats as sources for arthropod spillover into crop fields. This has important implications since it shows that landscape simplification through the loss of semi-natural habitats has negative consequences on arthropod biodiversity even in traditional farming landscapes with small fields and high crop diversity.

In certain scenarios, abandoned almond orchards could increase landscape heterogeneity and complexity by providing a different habitat for beneficial arthropods. However, traditional almond orchards harbor unique spider and parasitoid communities that, including bees, are equally as rich and abundant as (or even more abundant than) those of the more complex abandoned orchards for certain periods. The biodiversity associated with these orchards relies on their traditional management to subsist, and although an initial increase in complexity and biodiversity is observed after abandonment, theory and empirical studies predict a decrease in both after the vegetation succession has reached the state of the surrounding natural and semi-natural habitats (see [11,16], for reviews; however, see [18]).

Additionally, our findings are highly dependent on seasonality, emphasizing the relevance and suitability of including this factor in studies in regions with a Mediterranean climate since it constitutes an important driver of differences with studies in other regions.

More studies are needed with longer sampling periods and at different regions, especially including samplings in semi-natural habitats, to assess the extent to which abandoned orchards could support similar arthropod communities. Assuming the temporal and spatial limitations of our study, we consider the suitability of working at a regional scale to adapt the potential recommendations to a specific context, taking into account the importance of the seasonality and the different cultural and socioeconomic conditioning factors within each region.

From a conservation management perspective, efforts should be made to halt the abandonment progress, maintaining traditional almond orchards and conserving semi-natural habitats since both provide complementary habitats for beneficial arthropods, to protect the rich biodiversity associated with these traditional cultural landscapes.

## Figures and Tables

**Table 1:** Results of the PERMANOVA for the variables *system* (abandoned vs. traditional), *sampling month*, and *level of semi-natural habitats* (high vs. low) (full model results and pairwise comparisons). SNH = semi-natural habitats.

Response Variable	Explanatory Variables	d.f.	Pseudo-F	*p*-Value
Spiders	*System*	1	5.761	0.001
	*Sampling month*	3	10.754	0.001
	*System: Sampling month*	3	2.114	0.003
Parasitoids	*System*	1	1.549	0.177
	*Sampling month*	3	12.696	0.001
	*System: Sampling month*	3	2.351	0.018
Bees	*Sampling month*	3	13.263	0.001
	*Level of SNH*	1	2.242	0.041
	*Sampling month: Level of SNH*	3	1.466	0.103
Pairwise comparisons			Pseudo-t	*p*-value
Spiders	Traditional—Abandoned, February = 0		2.102	0.002
	Traditional—Abandoned, May = 0		1.295	0.100
	Traditional—Abandoned, July = 0		1.773	0.023
	Traditional—Abandoned, September = 0		1.987	0.004
Parasitoids	Traditional—Abandoned, February = 0		0.835	0.641
	Traditional—Abandoned, May = 0		1.830	0.025
	Traditional—Abandoned, July = 0		1.221	0.171
	Traditional—Abandoned, September = 0		2.009	0.001
Bees	Low—High, February = 0		1.998	0.018
	Low—High, May = 0		1.143	0.283
	Low—High, July = 0		0.974	0.477
	Low—High, September = 0		0.653	0.664

## Data Availability

The data presented in this study are openly available in Mendeley Data at https://doi.org/10.17632/2nnxg4w39s.1.

## Supplementary Materials

The following supporting information can be downloaded at: https://www.mdpi.com/article/10.3390/insects14030277/s1, Table S1: Complete results of the different models for the richness and abundance of spiders, parasitoids, and bees with the variables *system*, *sampling month* and *level of semi-natural habitats*; Table S2: Complete results of the different models for the richness and abundance of spiders, parasitoids, and bees with the variables *system* and *percentage of semi-natural habitats* at 150 m and 500 m; Table S3: Complete results of the different models for the abundance of the most dominant families or genera with the variables *system*, *sampling month* and *level of semi-natural habitats*; Table S4: Complete results of the different models for the abundance of the most dominant families or genera with the variables *system* and *percentage of semi-natural habitats* at 150 m and 500 m.
