# Supplementary material for: Effects of Traditional Orchard Abandonment and Landscape Context on the Beneficial Arthropod Community in a Mediterranean Agroecosystem
Source: Insects. 2023 Mar 10;14(3):277. doi: 10.3390/insects14030277 (PMC10056667; doi:10.3390/insects14030277)
Supplement: Supplementary file 1 [file insects-14-00277-s001.zip › insects-2229777-supplementary.pdf]

Table S1. Complete results of the different models for the richness and abundance of spiders, parasitoids, and bees. Estimates, standard errors, test statistics, p-values, and significance levels (ns > 0.1, · < 0.1, * < 0.05, ** < 0.01, and *** < 0.001) for the intercept and the explanatory variables s*ystem*, the *sampling month*, *level of semi-natural habitats* (SNH), and their interactions (when significant) are provided. In the first column, the type of model (LME, linear mixed model; GLMM, generalized linear mixed model), the transformation applied on the explanatory variable (if any), and the variance structure added to the model (if any) are given.

Response variable	Explanatory variable	Value / Estimate	Std. error	t-value / z-value	p-value	*
Spider family richness (LME) (varIdent(form =~ 1 ǀ *Sampling month*), varIdent(form=~ 1 ǀ *System*))	(Intercept)	11.879	0.469	25.349	0.000	***
	System (Traditional)	-1.662	0.522	-3.185	0.010	*
	Sampling month (May)	0.971	0.612	1.587	0.122	ns
	Sampling month (July)	3.323	0.749	4.439	0.000	***
	Sampling month (September)	0.736	0.595	1.236	0.225	ns
Spider abundance (GLMM – negative binomial)	(Intercept)	4.219	0.134	31.380	<0.001	***
	System (Traditional)	0.035	0.188	0.186	0.852	ns
	Sampling month (May)	0.274	0.178	1.539	0.124	ns
	Sampling month (July)	1.006	0.157	6.386	<0.001	***
	Sampling month (September)	0.473	0.171	2.760	0.006	**
	System (Traditional) : *Sampling month* (May)	0.207	0.245	0.843	0.399	ns
	System (Traditional) : *Sampling month* (July)	-0.471	0.229	-2.056	0.040	*
	System (Traditional) : *Sampling month* (September)	-0.697	0.262	-2.665	0.008	**
	Pairwise					
	Abandoned – Traditional, February == 0	-0.035	0.188	-0.186	0.853	ns
	Abandoned – Traditional, May == 0	-0.242	0.158	-1.536	0.133	ns
	Abandoned – Traditional, July == 0	0.436	0.131	3.327	0.002	**
	Abandoned – Traditional, September == 0	0.662	0.182	3.642	<0.001	***
Parasitoid family richness (LME) (Square root) (varIdent (form=~1ǀ *Sampling month*)	(Intercept)	2.853	0.183	15.572	<0.001	***
	System (Traditional)	-0.024	0.259	-0.093	0.928	ns
	Sampling month (May)	0.714	0.184	3.877	0.001	**
	Sampling month (July)	0.681	0.231	2.942	0.006	**
	Sampling month (September)	0.183	0.249	0.736	0.468	ns
	Level of SNH (Low)	-0.214	0.080	-2.666	0.029	*
	System (Traditional) : *Sampling month* (May)	0.077	0.260	0.296	0.769	ns
	System (Traditional) : *Sampling month* (July)	-0.369	0.327	-1.128	0.268	ns
	System (Traditional) : *Sampling month* (September)	-0.590	0.352	-1.678	0.104	ns
	System (Traditional) : *Level of SNH* (Low)	0.274	0.114	2.408	0.043	*
	Pairwise					
	Abandoned – Traditional, February == 0	-0.113	0.253	-0.446	0.667	ns

	Abandoned – Traditional, May == 0	-0.190	0.063	-3.022	0.0165	*
	Abandoned – Traditional, July == 0	0.257	0.208	1.234	0.252	ns
	Abandoned – Traditional, September == 0	0.478	0.245	1.953	0.087	·
	Abandoned – Traditional, High == 0	0.245	0.118	2.076	0.072	·
	Abandoned – Traditional, Low == 0	-0.029	0.118	-0.247	0.811	ns
	High – Low, Abandoned == 0	0.215	0.081	2.666	0.029	*
	High – Low, Traditional == 0	-0.060	0.081	-0.739	0.481	ns
Parasitoid abundance (LME) (varIdent (form=~1 | *Sampling month*)	(Intercept)	24.167	8.528	2.834	0.009	**
	System (Traditional)	10.333	9.847	1.049	0.321	ns
	Sampling month (May)	86.833	18.344	4.734	<0.001	***
	Sampling month (July)	56.500	21.004	2.690	0.012	*
	Sampling month (September)	13.917	10.934	1.273	0.214	ns
	Level of SNH (Low)	-11.667	9.847	-1.185	0.266	ns
	System (Traditional) : *Sampling month* (May)	91.000	21.182	4.296	<0.001	***
	System (Traditional) : *Sampling month* (July)	-17.000	24.253	-0.701	0.489	ns
	System (Traditional) : *Sampling month* (September)	-30.500	12.626	-2.416	0.023	*
	Sampling month (May) : *Level of SNH* (Low)	-61.333	21.182	-2.896	0.007	**
	Sampling month (July) : *Level of SNH* (Low)	11.667	24.253	0.481	0.634	ns
	Sampling month (September) : *Level of SNH* (Low)	30.500	12.626	2.416	0.023	*
	Pairwise					
	Abandoned – Traditional, February == 0	-10.33	9.850	-1.049	0.321	ns
	Abandoned – Traditional, May == 0	-101.33	20.050	-5.055	<0.001	***
	Abandoned – Traditional, July == 0	6.670	23.27	0.287	0.781	ns
	Abandoned – Traditional, September == 0	20.170	10.61	1.900	0.090	·
	High – Low, February == 0	11.700	9.850	1.185	0.266	ns
	High – Low, May == 0	73.000	20.050	3.641	0.005	**
	High – Low, July == 0	0.000	23.270	0.000	1.000	ns
	High – Low, September == 0	-18.800	10.61	-1.775	0.110	ns
Bee genus richness (LME) (Square root)	(Intercept)	1.480	0.130	11.406	<0.001	***
	Sampling month (May)	0.506	0.158	3.205	0.003	**
	Sampling month (July)	0.737	0.158	4.670	<0.001	**
	Sampling month (September)	0.007	0.158	0.043	0.966	ns
Bee abundance (GLMM – poisson)	(Intercept)	3.485	0.195	17.832	<0.001	***
	Sampling month (May)	-0.966	0.129	-7.487	<0.001	***
	Sampling month (July)	-1.015	0.131	-7.729	<0.001	***
	Sampling month (September)	-2.551	0.252	-10.132	<0.001	***

	Level of SNH (Low)	-1.000	0.291	-3.441	<0.001	***
	Sampling month (May) : *Level of SNH* (Low)	0.783	0.212	3.699	<0.001	***
	Sampling month (July) : *Level of SNH* (Low)	0.962	0.209	4.61	<0.001	***
	Sampling month (September) : *Level of SNH* (Low)	1.884	0.318	5.92	<0.001	***
	Pairwise					
	High – Low, February == 0	0.999	0.291	3.441	0.001	**
	High – Low, May == 0	0.217	0.307	0.705	0.485	ns
	High – Low, July == 0	0.038	0.305	0.123	0.903	ns
	High – Low, September == 0	-0.884	0.388	-2.275	0.029	*

Table S2. Complete results of the different models for the richness and abundance of spiders, parasitoids, and bees. Estimates, standard errors, test statistics, p-values, and significance levels (ns > 0.1, · < 0.1, * < 0.05, ** < 0.01, and *** < 0.001) for the intercept and the explanatory variables s*ystem*, *% of semi-natural habitats* at 150m or 500m, and the interaction (when significant). In the first column, the type of model (GLS, generalized least square model; GLM, generalized linear model) and the variance structure added to the model (if any) are given.

Response variable	Explanatory variable	Value / Estimate	Std. error	t-value / z-value	p-value	*
Spider family richness (GLS) (varIdent (form=~1 | *System*)	(Intercept)	20.729	0.756	27.408	0.000	***
	System (Traditional)	-3.245	1.170	-2.775	0.022	*
	% Semi-natural 150 m	-0.040	0.017	-2.359	0.043	*
Spider abundance (GLM – negative binomial) (dispformula = ~system)	(Intercept)	6.280	0.049	127.120	<0.001	***
	System (Traditional)	-0.467	0.080	-5.840	<0.001	***
	% Semi-natural 150 m	-0.005	0.001	-3.960	<0.001	***
	System (Traditional) : *% Semi-natural 150 m*	0.010	0.003	3.730	<0.001	***
Parasitoid family richness (GLS)	Nothing significant					
Parasitoid abundance (GLM – negative binomial)	(Intercept)	5.438	0.207	26.312	<0.001	***
	System (Traditional)	-0.030	0.271	-0.111	0.911	ns
	% Semi-natural 500 m	-0.002	0.005	-0.346	0.729	ns
	System (Traditional) : *% Semi-natural 500 m*	0.011	0.006	1.829	0.068	·
Bee genus richness (GLS)	(Intercept)	12.073	0.897	13.461	0.000	***
	System (Traditional)	-4.853	1.047	-4.634	0.002	**
	% Semi-natural 150 m	-0.066	0.020	-3.236	0.012	*
	System (Traditional) : *% Semi-natural 150 m*	0.093	0.029	3.170	0.013	*
Bee abundance (GLS)	(Intercept)	32.831	13.668	2.402	0.037	*
	% Semi-natural 500 m	0.601	0.319	1.884	0.089	·

Table S3. Complete results of the different models for the abundance of the most dominant families or genera. Estimates, standard errors, test statistics, p-values, and significance levels (ns > 0.1, · < 0.1, * < 0.05, ** < 0.01, and *** < 0.001) for the intercept and the explanatory variables s*ystem*, the *sampling month*, *level of semi-natural habitats* (SNH), and their interactions (when significant) are provided. In the first column, the type of model (LME, linear mixed model; GLMM, generalized linear mixed model), the transformation applied on the explanatory variable (if any), and the correlation or variance structure added to the model (if any) are given.

Response variable	Explanatory variable	Value / Estimate	Std. error	t-value / z-value	p-value	*
Araneidae abundance (GLMM – poisson)	(Intercept)	2.291	0.194	11.806	<0.001	***
	System (Traditional)	-0.407	0.178	-2.290	0.022	*
	Sampling month (May)	0.094	0.194	0.483	0.629	ns
	Sampling month (July)	0.487	0.178	2.737	0.006	**
	Sampling month (September)	-0.936	0.264	-3.548	<0.001	***
	Level of SNH (Low)	-0.430	0.268	-1.603	0.109	ns
	Sampling month (May) : *Level of SNH* (Low)	0.584	0.288	2.026	0.043	*
	Sampling month (July) : *Level of SNH* (Low)	0.435	0.272	1.6	0.110	ns
	Sampling month (September) : *Level of SNH* (Low)	0.874	0.364	2.403	0.016	*
	Pairwise					
	High – Low, February == 0	0.430	0.268	1.603	0.117	ns
	High – Low, May == 0	-0.155	0.235	-0.658	0.514	ns
	High – Low, July == 0	-0.006	0.215	-0.026	0.979	ns
	High – Low, September == 0	-0.444	0.323	-1.375	0.177	ns
Gnaphosidae abundance (GLMM – poisson)	(Intercept)	0.978	0.243	4.025	<0.001	***
	System (Traditional)	0.198	0.253	0.783	0.434	ns
	Sampling month (May)	1.379	0.189	7.292	<0.001	***
	Sampling month (July)	0.933	0.200	4.678	<0.001	***
	Sampling month (September)	0.337	0.221	1.520	0.1284	ns
	Level of SNH (Low)	-0.594	0.279	-2.13	0.033	*
	System (Traditional) : *Level of SNH* (Low)	0.796	0.370	2.15	0.032	*
	Pairwise					
	Abandoned – Traditional, High == 0	-0.198	0.253	-0.783	0.4383	ns
	Abandoned – Traditional, Low == 0	-0.994	0.270	-3.678	<0.001	***
	High – Low, Abandoned == 0	0.594	0.279	2.130	0.039	*
	High – Low, Traditional == 0	-0.202	0.243	-0.829	0.412	ns
Linyphiidae abundance (GLMM – poisson)	(Intercept)	2.094	0.242	8.653	<0.001	***
	System (Traditional)	0.442	0.268	1.649	0.099	·
	Sampling month (May)	0.410	0.221	1.856	0.063	·
	Sampling month (July)	0.113	0.231	0.491	0.624	ns
	Sampling month (September)	-0.196	0.259	-0.756	0.450	ns
	Level of SNH (Low)	-0.042	0.266	-0.157	0.875	ns
	System (Traditional) : *Sampling month* (May)	0.915	0.234	3.908	<0.001	***
	System (Traditional) : *Sampling month* (July)	-0.336	0.240	-1.405	0.160	ns

	System (Traditional) : *Sampling month* (September)	-0.590	0.283	-2.080	0.038	*
	Sampling month (May) : *Level of SNH* (Low)	-0.595	0.219	-2.714	0.007	**
	Sampling month (July) : *Level of SNH* (Low)	0.603	0.240	2.514	0.012	*
	Sampling month (September) : *Level of SNH* (Low)	0.200	0.282	0.710	0.478	ns
	Pairwise					
	Abandoned – Traditional, February == 0	-0.442	0.268	-1.649	0.108	ns
	Abandoned – Traditional, May == 0	-1.357	0.247	-5.500	<0.001	***
	Abandoned – Traditional, July == 0	-0.105	0.252	-0.417	0.679	ns
	Abandoned – Traditional, September == 0	0.148	0.294	0.503	0.618	ns
	High – Low, February == 0	0.042	0.266	0.157	0.876	ns
	High – Low, May == 0	0.6364	0.238	2.674	0.011	*
	High – Low, July == 0	-0.562	0.255	-2.204	0.034	*
	High – Low, September == 0	-0.158	0.294	-0.539	0.593	ns
Lycosidae abundance (GLMM – negative binomial)	(Intercept)	1.710	0.465	3.678	<0.001	***
	System (Traditional)	1.537	0.512	3.001	0.003	**
	Sampling month (May)	1.013	0.521	1.945	0.052	·
	Sampling month (July)	-0.536	0.565	-0.948	0.343	ns
	Sampling month (September)	-1.169	0.677	-1.726	0.084	·
	System (Traditional) : *Sampling month* (May)	-1.378	0.618	-2.230	0.026	*
	System (Traditional) : *Sampling month* (July)	-1.198	0.732	-1.637	0.102	ns
	System (Traditional) : *Sampling month* (September)	-2.720	1.243	-2.189	0.029	*
	Pairwise					
	Abandoned – Traditional, February == 0	-1.537	0.512	-3.001	0.005	**
	Abandoned – Traditional, May == 0	-0.159	0.431	-0.369	0.714	ns
	Abandoned – Traditional, July == 0	-0.339	0.614	-0.553	0.584	ns
	Abandoned – Traditional, September == 0	1.183	1.170	1.011	0.318	ns
Oxyopidae abundance (GLMM – poisson)	(Intercept)	3.000	0.218	13.745	<0.001	***
	System (Traditional)	-1.756	0.312	-5.633	<0.001	***
	Sampling month (May)	-1.119	0.227	-4.923	<0.001	***
	Sampling month (July)	1.232	0.139	8.883	<0.001	***
	Sampling month (September)	0.555	0.152	3.647	0.000	***
	Level of SNH (Low)	-0.119	0.272	-0.437	0.662	ns
	System (Traditional) : *Sampling month* (May)	2.076	0.313	6.630	<0.001	***
	System (Traditional) : *Sampling month* (July)	0.851	0.249	3.415	<0.001	***

	System (Traditional) : *Sampling month* (September)	0.465	0.265	1.759	0.079	·
	Sampling month (May) : *Level of SNH* (Low)	-0.012	0.274	-0.042	0.966	ns
	Sampling month (July) : *Level of SNH* (Low)	0.030	0.190	0.159	0.874	ns
	Sampling month (September) : *Level of SNH* (Low)	0.614	0.201	3.049	0.002	**
	Pairwise					
	Abandoned – Traditional, February == 0	1.756	0.312	5.633	<0.001	***
	Abandoned – Traditional, May == 0	-0.320	0.295	-1.083	0.286	ns
	Abandoned – Traditional, July == 0	0.905	0.226	4.001	<0.001	***
	Abandoned – Traditional, September == 0	1.291	0.242	5.327	<0.001	***
	High – Low, February == 0	0.119	0.272	0.437	0.665	ns
	High – Low, May == 0	0.130	0.293	0.445	0.659	ns
	High – Low, July == 0	0.089	0.225	0.394	0.696	ns
	High – Low, September == 0	-0.495	0.236	-2.099	0.043	*
Philodromidae abundance (GLMM – negative binomial)	(Intercept)	1.833	0.186	9.866	<0.001	***
	System (Traditional)	-0.254	0.133	-1.909	0.056	·
	Sampling month (May)	0.029	0.244	0.118	0.906	ns
	Sampling month (July)	1.157	0.202	5.725	<0.001	***
	Sampling month (September)	0.669	0.216	3.101	0.002	**
Salcitidae (GLMM – poisson)	(Intercept)	0.668	0.230	2.899	0.004	**
	System (Traditional)	-0.344	0.134	-2.565	0.010	*
	Sampling month (May)	0.095	0.309	0.308	0.758	ns
	Sampling month (July)	2.001	0.238	8.401	<0.001	***
	Sampling month (September)	0.668	0.275	2.428	0.015	*
Theridiidae (GLMM – poisson)	(Intercept)	1.065	0.325	3.273	0.001	**
	System (Traditional)	0.124	0.404	0.308	0.758	ns
	Sampling month (May)	-0.229	0.424	-0.540	0.589	ns
	Sampling month (July)	1.168	0.340	3.438	<0.001	***
	Sampling month (September)	0.983	0.356	2.761	0.006	**
	Level of SNH (Low)	0.308	0.398	0.774	0.439	ns
	System (Traditional) : *Sampling month* (May)	-0.304	0.448	-0.679	0.497	ns
	System (Traditional) : *Sampling month* (July)	-0.743	0.413	-1.799	0.072	·
	System (Traditional) : *Sampling month* (September)	-1.306	0.490	-2.667	0.008	**
	Sampling month (May) : *Level of SNH* (Low)	1.097	0.461	2.381	0.017	*
	Sampling month (July) : *Level of SNH* (Low)	-0.214	0.401	-0.535	0.593	ns
	Sampling month (September) : *Level of SNH* (Low)	-0.627	0.447	-1.402	0.161	ns
	System (Traditional) : *Level of SNH* (Low)	-0.690	0.400	-1.727	0.084	·

	Pairwise					
	Abandoned – Traditional, February == 0	0.221	0.354	0.623	0.537	ns
	Abandoned – Traditional, May == 0	0.525	0.325	1.615	0.116	ns
	Abandoned – Traditional, July == 0	0.963	0.276	3.487	0.001	**
	Abandoned – Traditional, September == 0	1.526	0.382	3.992	<0.001	***
	High – Low, February == 0	0.037	0.353	0.104	0.918	ns
	High – Low, May == 0	-1.060	0.346	-3.063	0.004	**
	High – Low, July == 0	0.251	0.264	0.952	0.348	ns
	High – Low, September == 0	0.664	0.332	1.999	0.054	·
	Abandoned – Traditional, High == 0	0.464	0.277	1.673	0.104	ns
	Abandoned – Traditional, Low == 0	1.154	0.286	4.038	<0.001	***
	High – Low, Abandoned == 0	-0.372	0.250	-1.489	0.146	ns
	High – Low, Traditional == 0	0.318	0.312	1.017	0.316	ns
Thomisidae abundance	(Intercept)	1.927	0.242	7.958	<0.001	***
	Sampling month (May)	0.516	0.309	1.671	0.095	·
	Sampling month (July)	-0.202	0.362	-0.558	0.577	ns
	Sampling month (September)	-0.545	0.402	-1.356	0.175	ns
	Level of SNH (Low)	-0.474	0.380	-1.248	0.212	ns
	Sampling month (May) : *Level of SNH* (Low)	-0.157	0.495	-0.317	0.751	ns
	Sampling month (July) : *Level of SNH* (Low)	0.766	0.523	1.464	0.143	ns
	Sampling month (September) : *Level of SNH* (Low)	1.079	0.554	1.950	0.051	·
	Pairwise					
	High – Low, February == 0	0.474	0.380	1.248	0.220	ns
	High – Low, May == 0	0.631	0.319	1.976	0.055	·
	High – Low, July == 0	-0.292	0.360	-0.811	0.422	ns
	High – Low, September == 0	-0.605	0.402	-1.504	0.141	ns
Zodariidae abundance (GLMM – poisson)	(Intercept)	-0.263	0.499	-0.527	0.598	ns
	Sampling month (May)	0.981	0.479	2.049	0.041	*
	Sampling month (July)	2.351	0.427	5.504	0.000	***
	Sampling month (September)	2.197	0.430	5.106	0.000	***
	Level of SNH (Low)	-1.717	1.153	-1.489	0.137	ns
	Sampling month (May) : *Level of SNH* (Low)	2.315	1.125	2.057	0.040	*
	Sampling month (July) : *Level of SNH* (Low)	1.966	1.094	1.798	0.072	·
	Sampling month (September) : *Level of SNH* (Low)	1.440	1.101	1.309	0.191	ns
	Pairwise					
	High – Low, February == 0	1.717	1.153	1.489	0.145	ns
	High – Low, May == 0	-0.598	0.513	-1.165	0.251	ns
	High – Low, July == 0	-0.249	0.439	-0.567	0.574	ns

	High – Low, September == 0	0.277	0.456	0.607	0.547	ns
Braconidae abundance (GLMM – poisson)	(Intercept)	1.808	0.256	7.059	<0.001	***
	System (Traditional)	-0.122	0.321	-0.380	0.704	ns
	Sampling month (May)	-0.032	0.293	-0.108	0.914	ns
	Sampling month (July)	-2.082	0.537	-3.876	<0.001	***
	Sampling month (September)	-1.998	0.628	-3.180	0.001	**
	Level of SNH (Low)	-0.687	0.332	-2.072	0.038	*
	System (Traditional) : *Sampling month* (May)	0.964	0.358	2.695	0.007	**
	System (Traditional) : *Sampling month* (July)	0.548	0.539	1.017	0.309	ns
	System (Traditional) : *Sampling month* (September)	-1.802	1.106	-1.630	0.103	ns
	Sampling month (May) : *Level of SNH* (Low)	-0.126	0.374	-0.336	0.737	ns
	Sampling month (July) : *Level of SNH* (Low)	1.538	0.568	2.709	0.007	**
	Sampling month (September) : *Level of SNH* (Low)	1.209	0.789	1.532	0.126	ns
	Pairwise					
	Abandoned – Traditional, February == 0	0.122	0.321	0.380	0.707	ns
	Abandoned – Traditional, May == 0	-0.842	0.287	-2.931	0.006	**
	Abandoned – Traditional, July == 0	-0.426	0.487	-0.875	0.388	ns
	Abandoned – Traditional, September == 0	1.924	1.082	1.778	0.084	·
	High – Low, February == 0	0.687	0.332	2.072	0.046	*
	High – Low, May == 0	0.813	0.286	2.838	0.008	**
	High – Low, July == 0	-0.851	0.515	-1.653	0.107	ns
	High – Low, September == 0	-0.522	0.754	-0.593	0.493	ns
Encyrtidae abundance (GLMM – poisson) (dispformula = ~sampling month)	(Intercept)	-0.508	0.530	-0.959	0.338	ns
	System (Traditional)	-0.253	0.802	-0.316	0.752	ns
	Sampling month (May)	1.609	0.548	2.938	0.003	**
	Sampling month (July)	3.209	0.510	6.292	<0.001	***
	Sampling month (September)	1.910	0.536	3.564	<0.001	***
	System (Traditional) : *Sampling month* (May)	0.182	0.830	0.220	0.826	ns
	System (Traditional) : *Sampling month* (July)	-0.752	0.789	-0.954	0.340	ns
	System (Traditional) : *Sampling month* (September)	-0.443	0.835	-0.531	0.596	ns
	Pairwise					
	Abandoned – Traditional, February == 0	0.253	0.802	0.316	0.754	ns
	Abandoned – Traditional, May == 0	0.071	0.407	0.175	0.862	ns
	Abandoned – Traditional, July == 0	1.005	0.314	3.203	0.003	**

	Abandoned – Traditional, September == 0	0.697	0.417	1.671	0.103	ns
Eulophidae abundance (GLMM – poisson)	(Intercept)	0.042	0.553	0.076	0.939	ns
	System (Traditional)	0.130	0.652	0.200	0.842	ns
	Sampling month (May)	2.479	0.537	4.615	<0.001	***
	Sampling month (July)	2.842	0.528	5.383	<0.001	***
	Sampling month (September)	1.980	0.549	3.604	<0.001	***
	Level of SNH (Low)	-0.627	0.671	-0.934	0.350	ns
	System (Traditional) : *Sampling month* (May)	0.264	0.636	0.415	0.678	ns
	System (Traditional) : *Sampling month* (July)	-0.209	0.623	-0.336	0.737	ns
	System (Traditional) : *Sampling month* (September)	-1.370	0.649	-2.111	0.035	*
	Sampling month (May) : *Level of SNH* (Low)	-0.747	0.666	-1.121	0.262	ns
	Sampling month (July) : *Level of SNH* (Low)	0.598	0.643	0.930	0.353	ns
	Sampling month (September) : *Level of SNH* (Low)	1.681	0.665	2.525	0.012	*
	Pairwise					
	Abandoned – Traditional, February == 0	-0.130	0.652	-0.200	0.843	ns
	Abandoned – Traditional, May == 0	-0.394	0.304	-1.297	0.203	ns
	Abandoned – Traditional, July == 0	0.079	0.271	0.292	0.772	ns
	Abandoned – Traditional, September == 0	1.239	0.323	3.832	<0.001	***
	High – Low, February == 0	0.627	0.671	0.934	0.357	ns
	High – Low, May == 0	1.374	0.323	4.252	<0.001	***
	High – Low, July == 0	0.029	0.272	0.108	0.945	ns
	High – Low, September == 0	-1.054	0.321	-3.286	0.002	**
Mymaridae abundance (GLMM – poisson)	(Intercept)	-0.049	0.586	-0.084	0.933	ns
	System (Traditional)	2.098	0.638	3.288	0.001	**
	Sampling month (May)	2.866	0.518	5.538	<0.001	***
	Sampling month (July)	1.213	0.572	2.121	0.034	*
	Sampling month (September)	0.419	0.655	0.639	0.523	ns
	Level of SNH (Low)	-1.434	0.528	-2.716	0.007	**
	System (Traditional) : *Sampling month* (May)	-0.914	0.548	-1.668	0.095	·
	System (Traditional) : *Sampling month* (July)	-2.567	0.638	-4.021	<0.001	***
	System (Traditional) : *Sampling month* (September)	-3.575	0.791	-4.522	<0.001	***
	Sampling month (May) : *Level of SNH* (Low)	0.677	0.404	1.677	0.094	·
	Sampling month (July) : *Level of SNH* (Low)	1.449	0.527	2.750	0.006	**
	Sampling month (September) : *Level of SNH* (Low)	2.149	0.657	3.271	0.001	**

	Pairwise					
	Abandoned – Traditional, February == 0	-2.098	0.638	-3.288	0.002	**
	Abandoned – Traditional, May == 0	-1.183	0.369	-3.209	0.003	**
	Abandoned – Traditional, July == 0	0.469	0.485	0.968	0.340	ns
	Abandoned – Traditional, September == 0	1.477	0.668	2.211	0.034	*
	High – Low, February == 0	1.434	0.528	2.716	0.010	*
	High – Low, May == 0	0.757	0.369	2.055	0.047	*
	High – Low, July == 0	-0.015	0.482	-0.031	0.976	ns
	High – Low, September == 0	-0.714	0.613	-1.164	0.252	ns
Platygastridae abundance (GLMM – poisson)	(Intercept)	-1.215	0.742	-1.638	0.102	ns
	System (Traditional)	0.383	0.965	0.397	0.692	ns
	Sampling month (May)	3.277	0.720	4.550	<0.001	***
	Sampling month (July)	1.705	0.769	2.218	0.027	*
	Sampling month (September)	0.000	1.000	0.000	1.000	ns
	System (Traditional) : *Sampling month* (May)	-0.569	0.935	-0.609	0.543	ns
	System (Traditional) : *Sampling month* (July)	1.047	0.972	1.077	0.282	ns
	System (Traditional) : *Sampling month* (September)	2.428	1.167	2.080	0.038	*
	Pairwise					
	Abandoned – Traditional, February == 0	-0.383	0.965	-0.397	0.694	ns
	Abandoned – Traditional, May == 0	0.186	0.372	0.501	0.619	ns
	Abandoned – Traditional, July == 0	-1.430	0.458	-3.123	0.003	**
	Abandoned – Traditional, September == 0	-2.811	0.792	-3.550	0.001	**
Pteromalidae abundance (GLMM – negative binomial)	(Intercept)	0.729	0.383	1.903	0.057	·
	System (Traditional)	0.882	0.235	3.750	<0.001	***
	Sampling month (May)	1.651	0.380	4.345	<0.001	***
	Sampling month (July)	-0.194	0.507	-0.383	0.701	ns
	Sampling month (September)	-20.618	8643.590	-0.002	0.998	ns
	Level of SNH (Low)	-0.054	0.486	-0.111	0.911	ns
	Sampling month (May) : *Level of SNH* (Low)	-1.226	0.592	-2.071	0.038	*
	Sampling month (July) : *Level of SNH* (Low)	0.465	0.688	0.676	0.499	ns
	Sampling month (September) : *Level of SNH* (Low)	19.282	8643.590	0.002	0.998	ns
	Pairwise					
	High – Low, February == 0	0.054	0.486	0.111	0.912	ns
	High – Low, May == 0	1.280	0.337	3.795	<0.001	***
	High – Low, July == 0	-0.411	0.484	-0.849	0.401	ns
	High – Low, September == 0	-19.228	8643.590	-0.002	0.998	ns
	(Intercept)	1.539	0.279	5.513	<0.001	***

Scelionidae abundance (GLMM – poisson)	*System* (Traditional)	0.799	0.314	2.546	0.011	*
	Sampling month (May)	1.577	0.242	6.505	<0.001	***
	Sampling month (July)	1.782	0.239	7.454	<0.001	***
	Sampling month (September)	1.272	0.252	5.056	<0.001	***
	Level of SNH (Low)	-0.452	0.307	-1.472	0.141	ns
	System (Traditional) : *Sampling month* (May)	0.507	0.267	1.897	0.058	·
	System (Traditional) : *Sampling month* (July)	-0.701	0.268	-2.616	0.009	**
	System (Traditional) : *Sampling month* (September)	-1.182	0.288	-4.108	<0.001	***
	Sampling month (May) : *Level of SNH* (Low)	-0.348	0.254	-1.369	0.171	ns
	Sampling month (July) : *Level of SNH* (Low)	0.151	0.260	0.579	0.563	ns
	Sampling month (September) : *Level of SNH* (Low)	0.466	0.280	1.665	0.096	·
	Pairwise					
	Abandoned – Traditional, February == 0	-0.799	0.314	-2.546	0.016	*
	Abandoned – Traditional, May == 0	-1.306	0.227	-5.742	<0.001	***
	Abandoned – Traditional, July == 0	-0.099	0.227	-0.434	0.667	ns
	Abandoned – Traditional, September == 0	0.383	0.250	1.533	0.134	ns
	High – Low, February == 0	0.452	0.307	1.472	0.150	ns
	High – Low, May == 0	0.800	0.224	3.575	0.001	**
	High – Low, July == 0	0.301	0.227	1.323	0.195	ns
	High – Low, September == 0	-0.014	0.248	-0.057	0.955	ns
Andrena abundance (GLMM – poisson)	(Intercept)	2.035	0.232	8.755	<0.001	***
	System (Traditional)	-0.346	0.301	-1.151	0.250	ns
	Sampling month (May)	-0.710	0.313	-2.266	0.023	*
	Sampling month (July)	-3.333	1.039	-3.209	0.001	**
	Sampling month (September)	-22.367	14840.820	-0.002	0.999	ns
	Level of SNH (Low)	-0.714	0.310	-2.299	0.022	*
	System (Traditional) : *Sampling month* (May)	1.036	0.366	2.830	0.005	**
	System (Traditional) : *Sampling month* (July)	-1.198	1.133	-1.057	0.290	ns
	System (Traditional) : *Sampling month* (September)	-1.450	35128.136	0.000	1.000	ns
	Sampling month (May) : *Level of SNH* (Low)	0.759	0.369	2.060	0.039	*
	Sampling month (July) : *Level of SNH* (Low)	2.267	1.131	2.005	0.045	*
	Sampling month (September) : *Level of SNH* (Low)	-0.726	30942.871	0.000	1.000	ns
	Pairwise					
	Abandoned – Traditional, February == 0	0.346	0.300	1.151	0.258	ns

	Abandoned – Traditional, May == 0	-0.690	0.290	-2.381	0.023	*
	Abandoned – Traditional, July == 0	1.544	1.110	1.393	0.173	ns
	Abandoned – Traditional, September == 0	1.796	35128.14	0.000	1.000	ns
	High – Low, February == 0	0.714	0.310	2.299	0.028	*
	High – Low, May == 0	-0.046	0.279	-0.163	0.871	ns
	High – Low, July == 0	-1.553	1.110	-1.402	0.170	ns
	High – Low, September == 0	1.440	0.000	0.000	1.000	ns
Eucera abundance (GLMM – poisson)	(Intercept)	2.053	0.562	3.652	<0.001	***
	Sampling month (May)	-2.833	0.460	-6.157	0.000	***
	Sampling month (July)	-23.918	16943.241	-0.001	0.999	ns
	Sampling month (September)	-23.918	16943.241	-0.001	0.999	ns
	Level of SNH (Low)	-1.854	0.847	-2.189	0.029	*
	Sampling month (May) : *Level of SNH* (Low)	1.889	0.641	2.949	0.003	**
	Sampling month (July) : *Level of SNH* (Low)	-1.187	68773.714	0.000	1.000	ns
	Sampling month (September) : *Level of SNH* (Low)	-1.187	68773.714	0.000	1.000	ns
	Pairwise					
	High – Low, February == 0	1.854	0.850	2.189	0.035	*
	High – Low, May == 0	-0.035	1.000	-0.035	0.973	ns
	High – Low, July == 0	3.041	68773.71	0.000	1.000	ns
	High – Low, September == 0	3.041	68773.71	0.000	1.000	ns
Lasioglossum abundance (GLMM – poisson) (dispformula = ~sampling month)	(Intercept)	2.592	0.231	11.201	<0.001	***
	Sampling month (May)	-1.144	0.213	-5.363	<0.001	***
	Sampling month (July)	-0.985	0.201	-4.898	<0.001	***
	Sampling month (September)	-2.565	0.392	-6.539	<0.001	***
	Level of SNH (Low)	-0.865	0.350	-2.470	0.014	*
	Sampling month (May) : *Level of SNH* (Low)	-0.415	0.444	-0.935	0.350	ns
	Sampling month (July) : *Level of SNH* (Low)	0.482	0.332	1.453	0.146	ns
	Sampling month (September) : *Level of SNH* (Low)	1.125	0.540	2.084	0.037	*
	Pairwise					
	High – Low, February == 0	0.865	0.350	2.470	0.018	*
	High – Low, May == 0	1.279	0.495	2.586	0.014	*
	High – Low, July == 0	0.382	0.398	0.961	0.342	ns
	High – Low, September == 0	-0.260	0.582	-0.446	0.658	ns

Table S4. Complete results of the different models for the abundance of the most dominant families or genera. Estimates, standard errors, test statistics, p-values, and significance levels (ns > 0.1, · < 0.1, * < 0.05, ** < 0.01, and *** < 0.001) for the intercept and the explanatory variables system, % of semi-natural habitats at 150m or 500m, and the interaction (when significant. In the first column, the type of model (GLS, generalized least square model; GLM, generalized linear model) and the variance or correlation structures added to the model (if any) are given.

Response variable	Explanatory variable	Value / Estimate	Std. error	t-value / z-value	p-value	*
Araneidae abundance (GLS) (correlation = corGaus(form =~ UTMX + UTMY)	(Intercept)	80.175	10.304	7.781	<0.001	***
	System (Traditional)	-43.262	2.793	-15.491	<0.001	***
	% Semi-natural 150 m	-0.707	0.080	-8.861	<0.001	***
	System (Traditional) : *% Semi-natural 150 m*	0.664	0.125	5.305	<0.001	***
Gnaphosidae abundance (GLM – negative binomial) (dispformula = ~semi150)	(Intercept)	2.158	0.606	3.562	0.000	***
	System (Traditional)	1.480	0.628	2.358	0.018	*
	% Semi-natural 150 m	0.018	0.012	1.519	0.129	ns
	System (Traditional) : *% Semi-natural 150 m*	-0.028	0.014	-2.052	0.040	*
Linyphiidae abundance (GLM – negative binomial) (dispformula = ~ semi150)	(Intercept)	4.247	0.267	15.927	<0.001	***
	System (Traditional)	-0.143	0.309	-0.463	0.643	ns
	% Semi-natural 150 m	-0.015	0.008	-1.905	0.057	.
	System (Traditional) : *% Semi-natural 150 m*	0.025	0.010	2.416	0.016	*
Lycosidae abundance (GLM – negative binomial)	(Intercept)	3.280	0.331	9.922	<0.001	***
	System (Traditional)	0.686	0.387	1.771	0.077	·
Oxyopidae abundance (GLM – poisson) (gaussian correlation)	(Intercept)	4.957	0.269	18.416	<0.001	***
	System (Traditional)	-1.415	0.184	-7.687	<0.001	***
	% Semi-natural 500 m	0.000	0.005	-0.078	0.938	ns
	System (Traditional) : *% Semi-natural 500 m*	0.010	0.005	2.124	0.034	*
Philodromidae abundance (GLM – negative binomial)	(Intercept)	3.867	0.165	23.421	<0.001	***
	System (Traditional)	-0.597	0.256	-2.338	0.019	*
	% Semi-natural 500 m	-0.002	0.004	-0.472	0.637	ns
	System (Traditional) : *% Semi-natural 500 m*	0.010	0.006	1.672	0.095	·
Salticidae abundance (GLM – gaussian) (square root transformed, gaussian correlation)	(Intercept)	4.891	0.229	21.341	<0.001	***
	System (Traditional)	-0.883	0.068	-12.937	<0.001	***
	% Semi-natural 150 m	-0.005	0.002	-2.728	0.006	**
	System (Traditional) : *% Semi-natural 150 m*	-0.008	0.002	-3.188	0.001	**
Theridiidae abundance (GLM – poisson)	(Intercept)	3.435	0.163	21.059	<0.001	***
	System (Traditional)	-1.294	0.242	-5.355	<0.001	***
	% Semi-natural 150 m	-0.004	0.004	-1.128	0.259	ns
	System (Traditional) : *% Semi-natural 150 m*	0.020	0.007	2.852	0.004	**
Thomisidae abundance (GLM – poisson)	(Intercept)	3.602	0.152	23.723	<0.001	***
	System (Traditional)	-0.542	0.190	-2.856	0.004	**
	% Semi-natural 150 m	-0.006	0.004	-1.682	0.093	·

	System (Traditional) : % *Semi-natural 150 m*	0.013	0.005	2.336	0.019	*
Zodariidae abundance (GLM – negative binomial)	Nothing significant					
Braconidae abundance (GLM – gaussian) (dispformula = ~system)	(Intercept)	4.296	3.761	1.142	0.253	ns
	System (Traditional)	6.114	3.196	1.913	0.056	·
	% *Semi-natural 500 m*	0.189	0.063	2.981	0.003	**
Encyrtidae abundance (GLM – poisson) (gaussian correlation)	(Intercept)	3.954	0.423	9.344	<0.001	***
	System (Traditional)	-1.605	0.240	-6.694	<0.001	***
	% *Semi-natural 150 m*	-0.021	0.005	-4.237	<0.001	***
	System (Traditional) : % *Semi-natural 150 m*	0.020	0.008	2.599	0.009	**
Eulophidae abundance (GLM – gaussian) (log transformed)	(Intercept)	4.192	0.323	12.972	<0.001	***
	System (Traditional)	-1.019	0.377	-2.701	0.007	**
	% *Semi-natural 150 m*	-0.013	0.007	-1.739	0.082	·
	System (Traditional) : % *Semi-natural 150 m*	0.031	0.011	2.885	0.004	**
Mymaridae abundance (GLM – negative binomial)	(Intercept)	3.201	0.300	10.674	<0.001	***
	System (Traditional)	0.707	0.356	1.985	0.047	*
Platygastridae abundance (GLS)	(Intercept)	24.963	3.737	6.680	<0.001	***
	% *Semi-natural 150 m*	-0.308	0.104	-2.973	0.014	*
Pteromalidae abundance (GLS)	(Intercept)	9.899	4.448	2.225	0.057	·
	System (Traditional)	1.490	6.140	0.243	0.814	ns
	% *Semi-natural 500 m*	0.007	0.095	0.071	0.946	ns
	System (Traditional) : % *Semi-natural 500 m*	0.524	0.145	3.609	0.007	**
Scelionidae abundance (GLM – negative binomial)	(Intercept)	4.188	0.185	22.605	<0.001	***
	System (Traditional)	0.513	0.231	2.219	0.027	*
Andrena abundance (GLM – poisson) (dispformula = ~system)	(Intercept)	2.753	0.239	11.526	<0.001	***
	System (Traditional)	-0.359	0.287	-1.250	0.211	ns
	% *Semi-natural 150 m*	-0.010	0.006	-1.779	0.075	·
	System (Traditional) : % *Semi-natural 150 m*	0.016	0.008	1.990	0.047	*
Eucera abundance (GLS)	(Intercept)	-1.465	5.743	-0.255	0.804	ns
	% *Semi-natural 500 m*	0.306	0.134	2.287	0.045	*
Lasioglossum abundance (GLS)	(Intercept)	6.761	6.070	1.114	0.291	ns
	% *Semi-natural 500 m*	0.365	0.142	2.576	0.028	*
